# Supplementary material for: Studying Macromolecular Composition in Cell–Cell Interfaces Using 3D Membrane Reconstitution Systems
Source: Adv Sci (Weinh). 2026 Mar 24;13(31):e22443. doi: 10.1002/advs.202522443 (PMC13588059; doi:10.1002/advs.202522443)
Supplement: Supplementary file 1 [file ADVS-13-e22443-s001.pdf]

Supplement Information and Supplementary Figures

Studying macromolecular composition in cell-cell interfaces using 3D membrane reconstitution systems

Franziska Ragaller[1], Amelie Maribel Schneider[1], Ellen Sjule[1], Renhua Sun[2], Xiao Han[2], Luca Andronico[1], Edward Jenkins[3], Michael Dustin[3], Adnane Achour[2], Erdinc Sezgin[1]

[1] Science for Life Laboratory, Department of Women's and Children's Health, Karolinska Institutet, 17165 Solna, Sweden

[2] Science for Life Laboratory, Department of Medicine, Karolinska Institute & Division of Infectious Diseases, Karolinska University Hospital, 17165 Solna, Sweden.

[3] Kennedy Institute of Rheumatology, NDORMS, University of Oxford, Oxford, OX3 7FY, UK.

* Correspondence: erdinc.sezgin@ki.se

Supplement Table 1: Proteins used with their respective fluorescent label and buffer.

Protein	fluorescent label	buffer
CD2	none	PBS
	Alexa Fluor 488	
	Alexa Fluor 647	
CD58	none	
	Alexa Fluor 488	
	Alexa Fluor 647	
pMHC/gp33	Alexa Fluor 488	
P14 TCR	none	
pMHC/9V	Alexa Fluor 488	
pMHC/3P9V	Alexa Fluor 488	
1G4 TCR	none	
PD-1	Alexa Fluor 488	
PD-L1	Alexa Fluor 647	
CTLA-4	Alexa Fluor 488	
CD80	Alexa Fluor 647	
CD84	Alexa Fluor 647	
SLAM F6	Alexa Fluor 647	
Nkp30/NCR3	Alexa Fluor 488	
B7-H6	Alexa Fluor 647	
SIRPα	Alexa Fluor 647	
CD45 (non-AB)	Alexa Fluor 647	
CD43	Alexa Fluor 488	
MUC1	Alexa Fluor 488	
WGA	Alexa Fluor 647	HBSS
AAL	Alexa Fluor 647	10 mM HEPES<br>150 mM NaCl<br>0.1 mM $CaCl_2$
SNA	Alexa Fluor 647	
MAL-II	Alexa Fluor 647	

Supplement Table 2: Protein amounts used in different model systems.

	**artificial contact (GUV-GUV)**	**semi-artificial contact (GUV-cell)**
**single protein**	~2.5 pmol	~ 1.25 pmol
**dual protein**		
contact forming protein	~1.25 pmol	~0.63 pmol
potentially excluded protein	0.16 - 2.5 pmol	0.16 - 2.5 pmol

Artificial GUV-GUV contacts – Quantification

Image analysis and quantification was performed in Fiji (Schindelin et al., 2012) and is illustrated in Supplement Figure 1A. The original multi-channel image is split into the different channels. Thresholding on fluorescent channels was used to define areas of contact and individual GUVs. These areas were saved as ROIs (regions of interest). Additional manual selection of contact, GUV1 and GUV2 was performed ,and these regions were combined with ROIs obtained from thresholding using the "AND" function. This excludes intensity from potential bright aggregates or other GUVs not involved in contact formation in the image. The fluorescent intensity was measured for each region and background in each channel. After background subtraction the fluorescence intensity of the contact was normalized to the sum of intensities outside of the contact of GUV1 and GUV2 for each channel. The sum of intensities of the GUVs was set to 1. Therefore, normalized intensity values above 1 indicate enrichment, below 1 exclusion and around 1 neither enrichment nor exclusion from the contact. To ensure this image analysis and quantification is correct, we used GUVs incorporating the lipid dye TF-chol and added either CD58-AF647 or unlabelled CD2. CD2-CD58-mediated contact formation occurs as indicated by the enrichment of CD58 at the contact, whereas the values for TF-chol fluctuate around 1, verifying our analysis strategy (Supplement Figure 1B). The presented image analysis pipeline was followed and adapted for the semi-artificial system with unlabelled or Lectin-labelled cells. For cells expressing ZAP70mNG, the ZAP70mNG signal recruited to the contact site was normalized to the average overall intensity of ZAP70mNG in the cell.

Artificial GUV-GUV contacts – further controls

To ensure the artificial contact-formation system works properly, enriched signal and contact formation are indeed mediated by protein interactions and correct analysis is conducted, we performed a number of controls. The fluorescent signal observed on the GUVs stems from fluorescently labelled protein and not free dye as the dyes alone do not bind to the GUVs containing Ni-functionalized lipids (Supplement Figure 2A). Protein-less GUVs incorporating either TF-PE or ASR-PE as fluorescent lipid dyes sometimes form "contacts", however this is rare and caused by chance (Supplement Figure 2B). As the proteins get into close physical proximity upon binding and accumulating at the contact, we performed a FRET control and quantified the signal at the contact upon 488 nm excitation only in both the green (495-600 nm) and red channel (645-750 nm) and no FRET could be observed (Supplement Figure 2C). Of note, CD2-AF488 can be detected on primarily CD58-AF647 positive GUVs and vice versa (Supplement Figure 1C), which is also observed for other protein combinations. This is due to the nature of the protein reconstitution on GUVs in our system. Ni-lipids and His-tagged proteins do not from a covalent bond, but a chelation bond, so binding is reversible. As soon

as the two GUV populations decorated with different proteins A and B are added together, there is an exchange of the proteins between the GUV populations (Raghunath and Dyer, 2019). This is determined by the number of Ni-lipids on the GUV, the length of the His-tag of the protein and its position in respect to the protein, and the amount of protein bound to the surface (protein crowding) (Raghunath and Dyer, 2019). Also, the protein enrichment fluctuates due to heterogeneity in lipid distribution – some GUVs will have more or less than 2% Ni-lipid in their outer lipid layer, even though all GUVs were produced from the same lipid mixture. Further, the method of protein labelling cannot ensure only one fluorescent label per protein molecule, further contributing to different degrees of enrichment of 2 proteins at the same contact.

CD45 density estimation on GUVs compared to Jurkat T cells

We estimated the density of CD45 (normalized counts per μm^2) reconstituted on GUVs and on the Jurkat T cell surface via flow cytometry (Supplementary Figure 5). Thereby 1.25 pmol CD45 were reconstituted, corresponding to the 0.5x or 1x conditions in the artificial or semi-artificial model systems, respectively. GUVs were produced incorporating a volumetric dye (AF488), CD45 was reconstituted, and they were stained with a surface dye (ASR-PE) and anti-CD45 BV510. Jurkat T cells were stained with the surface dye and anti-CD45 BV510 antibody (Supplementary Figure 5A). Furthermore, we prepared unstained and single stained samples to confirm negligible spillover of each fluorochromes into off-target channels. In addition, cells stained with the membrane dye were analyzed in confocal microscopy to estimate their size distribution (Supplementary Figure 5C).

Gated GUVs were double positive for both volumetric and surface dye, confirming that they remained intact during flow cytometry experiments. Furthermore, the intensity ratio between volumetric and surface dyes scaled linearly with the vesicle's size confirming their proportionality (Supplementary Figure 5B,D). We then estimated the vesicles size for both Jurkat cells and GUVs from flow cytometry data. Given that the FCS signal of a vesicle in flow cytometry is proportional to the vesicle's cross section, we derived a multiplication factor α to convert the FSC signal from individual Jurkat cells to a corresponding cell size, according to Eq. 1:

$$\alpha = \frac{FSC_{mean}}{\pi(\frac{d_{mean}}{2})^2}$$

Where FSC_{mean} is the mean scattered intensity from cells in flow cytometry data and d_{mean} the mean diameter calculated from microscopy data.

Then we used the α factor to convert each i-th FSC signal in flow cytometry data into the corresponding size according to Eq. 2:

$$d_i = 2\sqrt{\frac{FSC_i}{\pi\alpha}}$$

As shown in Supplementary Figure 5E, Jurkat sizes calculated from microscopy and flow data yielded similar distributions, supporting the validity of using the α factor for FCS to size conversion. Assuming the same α factor for both GUVs and cells biological membranes, we converted individual FSC intensities from flow cytometry data to the corresponding GUVs sizes. Once we derived the size for each individual GUV and Jurkat cell, we normalized the corresponding CD45 intensity signal by the calculated surface area. The distribution of normalized CD45 signal shows an almost twice as high density of CD45 on the GUV (corresponding to the 0.5x or 1x conditions in the artificial or semi-artificial model systems, respectively) compared to the surface of Jurkat T cells (Supplementary Figure 5F).

Supplementary Figures

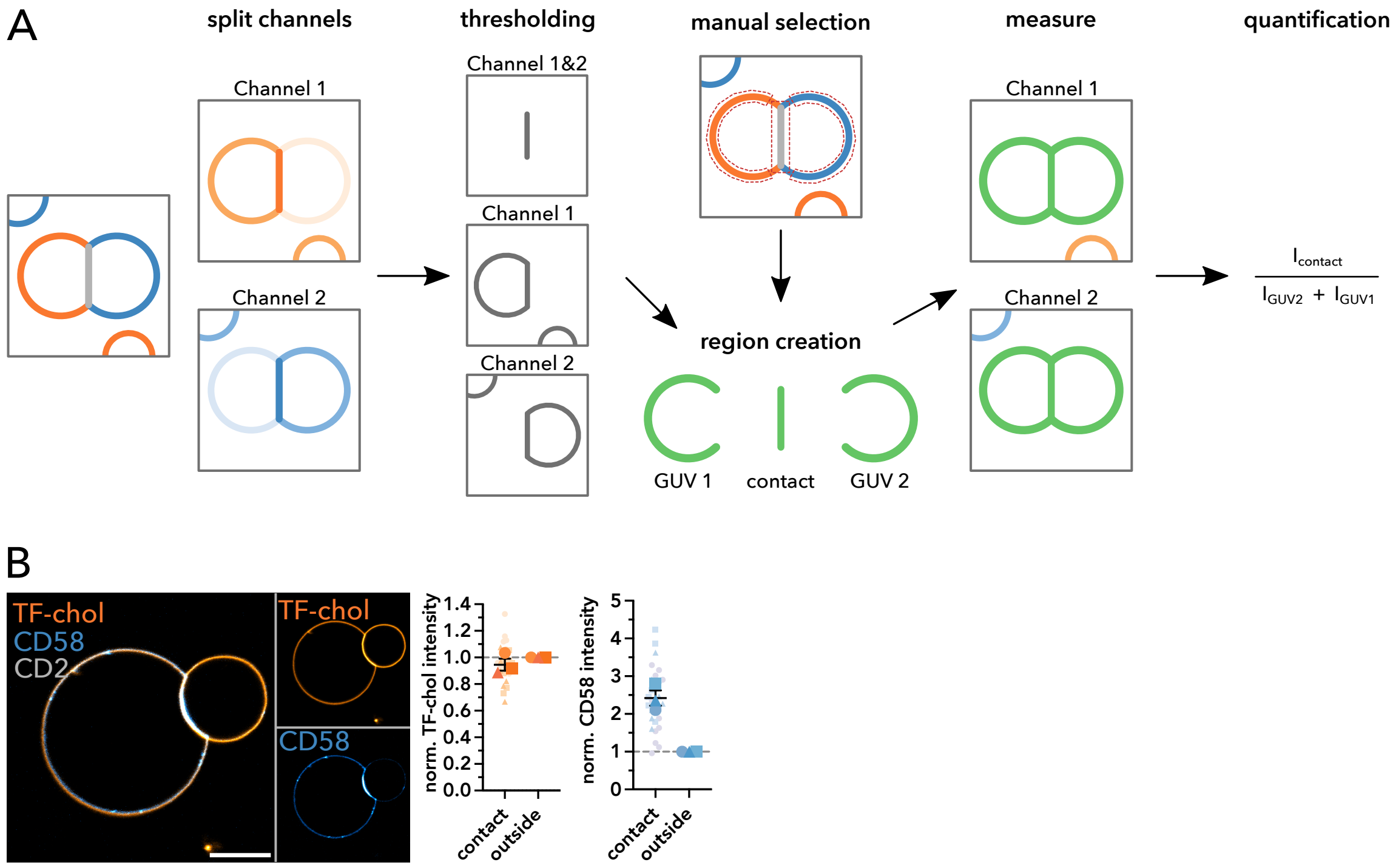

Supplement Figure 1: Fluorescent intensity quantification. A| Schematic representation of the image analysis using the artificial GUV-GUV system as an example. In Fiji, channels of the original image are split and the fluorescence channels are used for thresholding of the contact site and the individual GUVs. Additional manual selection of the areas is performed to ensure proper selection. The thresholded areas and manually selected areas are combined to create the regions: contact, GUV 1 and GUV 2. Fluorescence intensity in these regions is measured in each channel. After background subtraction the intensities are normalized and quantified. B| Quantification control. Representative confocal image of GUVs comprising Ni-lipid and 0.1% TF-chol decorated with either unlabeled CD2 or CD58-AF647 (left). TF-chol and CD58-AF647 intensity at the contact and outside was quantified and normalized (right).

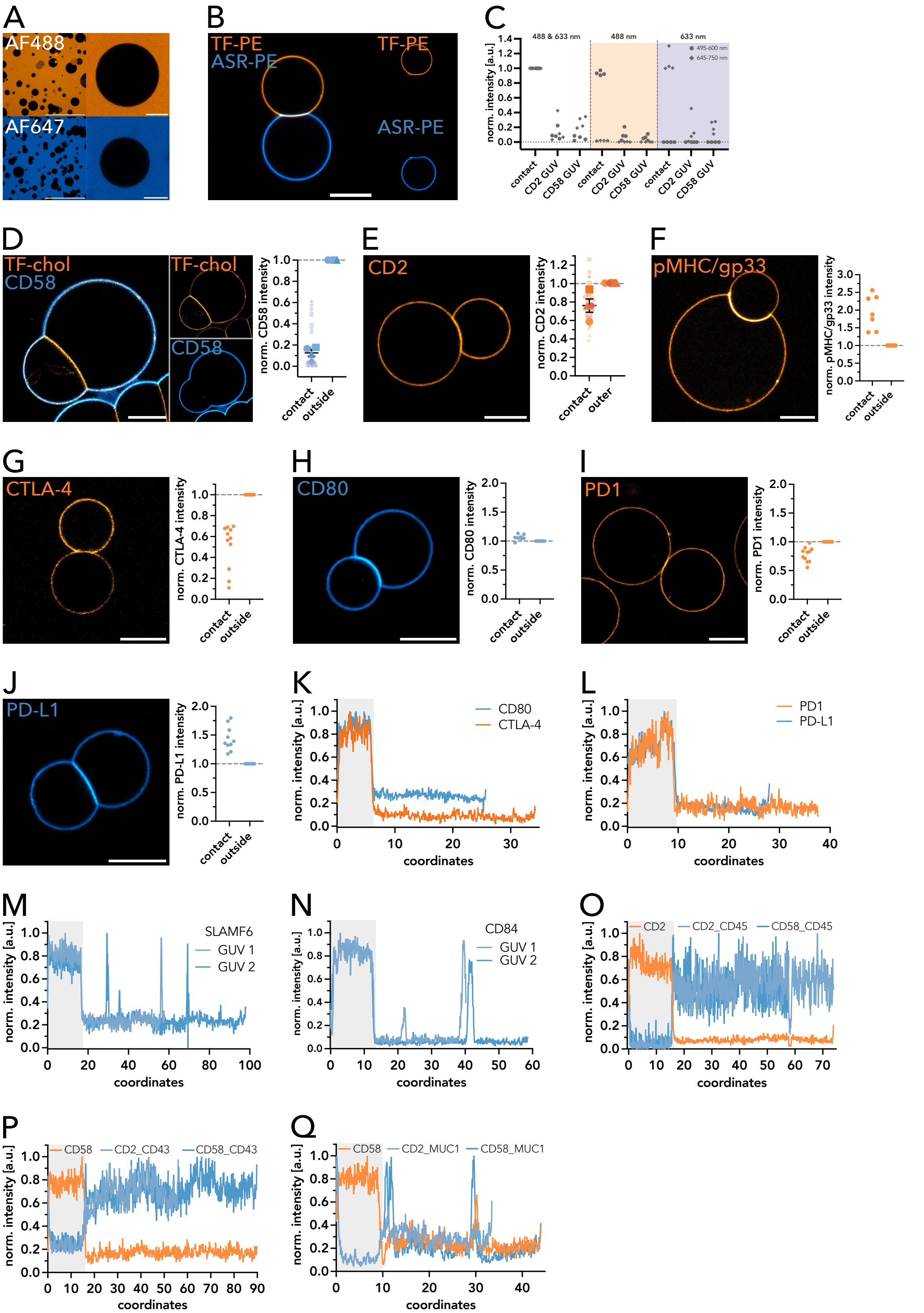

Supplement Figure 2: A| Alexa Fluor 488 and Alexa Fluor 647 dye control. Confocal images of GUVs comprising Ni-lipid and added fluorescent dye (1:10000). B| Contact formation control. Representative confocal image of GUVs comprising Ni-lipid and fluorescently labelled

lipids (0.1% ASR-PE or 0.01% TF-PE). C| FRET control. Depicted are normalized intensities of CD2-AF488 and CD58-AF647 at the contact and outside collected in two channels (dot: 495-600 nm; diamond: 645-750 nm) with both excitation lasers, only 488 nm excitation (light orange) and only 633 nm excitation (light blue). D| CD58-AF647 only control. Representative confocal image of GUVs comprising Ni-lipid and 0.1% TF-chol decorated with CD58-AF647 (left). CD58-AF647 intensity at the potential contact and outside was quantified and normalized (right). E-J| Single protein controls. Representative confocal images of GUVs comprising Ni-lipid and with CD2-AF488 (E), pMHC/gp33-AF488 (F), CTLA-4-AF88 (G), CD80-AF647 (H), PD1-AF488 (I), PD-L1-AF647 (J) (left). Fluorescent intensity at the potential contact and outside was quantified and normalized (right). K-L| Linearized fluorescence intensity profiles of the GUVs decorated with either CTLA-4-AF488 and CD80-AF647 (K) or PD-1-AF488 and PD-L1-AF647 (L) with the contact highlighted in grey in the confocal images of Figure 1 D-E. M-N| Linearized fluorescence intensity profiles of the GUVs decorated with either and SLAMF6-AF647 (M) or CD84-AF647 (N) with the contact highlighted in grey in the confocal images of Figure 1 F-G. O-Q| Linearized fluorescence intensity profiles of the GUVs decorated with CD2 and CD58 and additional CD45-AF647 (O), CD43-AF488 (P) or MUC1-AF488 (Q) with the contact highlighted in grey in the confocal images of Figure 1 H-J. Superplots show individual contacts as small symbols. Large symbols depict the mean of the individual biological replicates. Symbol corresponds to the individual biological replicate ($n \geq 3$). Standard error of the mean is shown. Scale bar corresponds to 10 μm (small field of view; single GUVs) or to 100 μm (large field of view; A).

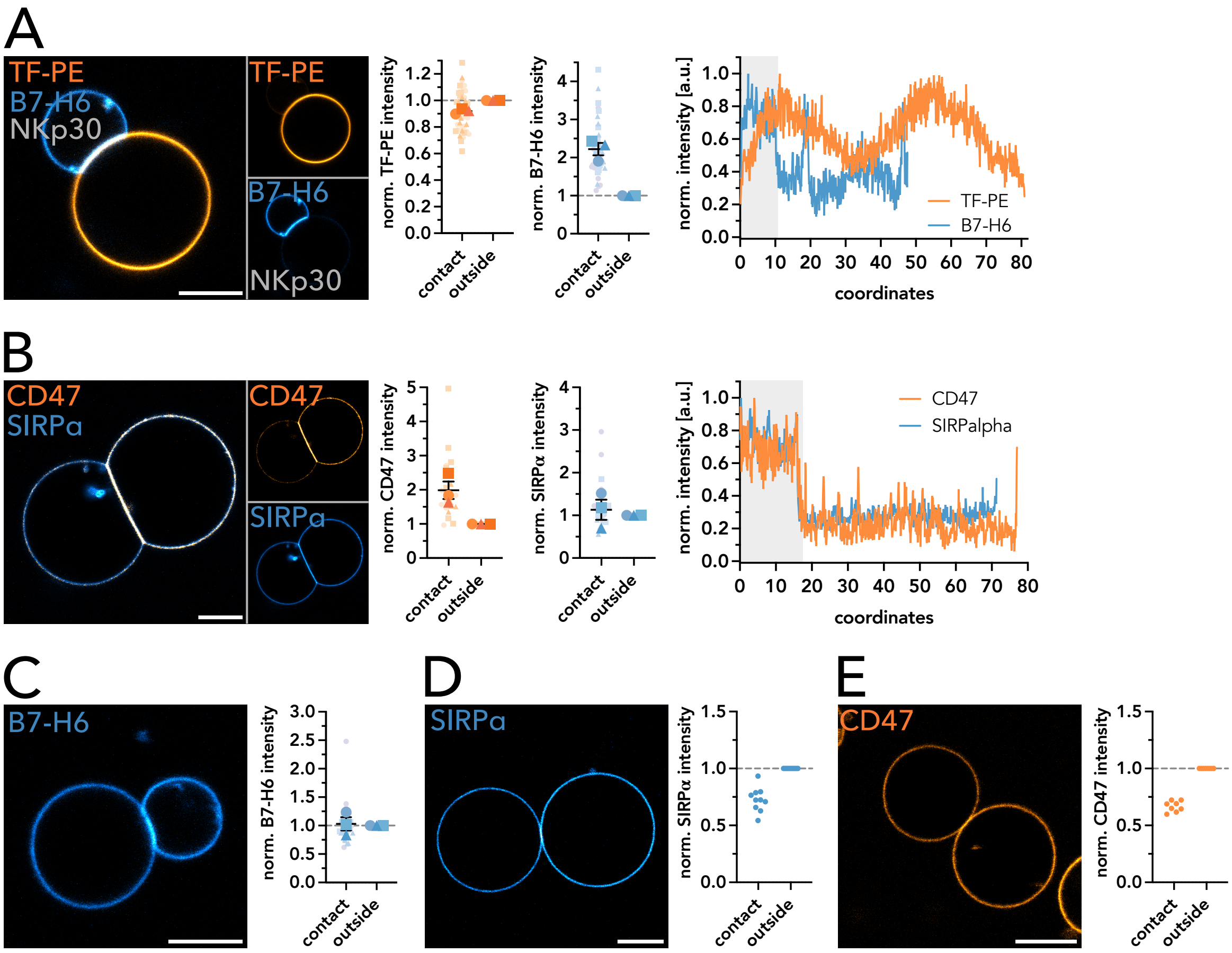

Supplement Figure 3: A| Representative confocal image of Nkp30-B7H6 contact formation (left). Nkp30 has no fluorescent label and was reconstituted on GUV incorporating TF-PE. TF-PE intensity and B7H6-AF647 at the contact and outside was quantified and normalized (middle). Linearized fluorescence intensity profiles of the GUVs with the contact highlighted in grey in the confocal image (right). B| Representative confocal image of CD47-SIRPα contact formation (left). CD47-AF488 and SIRPα-AF647 intensity at the contact and outside was quantified and normalized (middle). Linearized fluorescence intensity profiles of the GUVs with the contact highlighted in grey in the confocal image (right). C-E| Single protein controls. Representative confocal images of GUVs comprising Ni-lipid and with B7-H6-AF647 (C), SIRPα-AF647 (D) and CD47-AF488 (E) (left). Fluorescent intensity at the potential contact and outside was quantified and normalized (right). Superplots show individual contacts as small symbols. Large symbols depict the mean of the individual biological replicates. Symbol corresponds to the individual biological replicate (n=3). Standard error of the mean is shown. Scale bar corresponds to 10 µm.

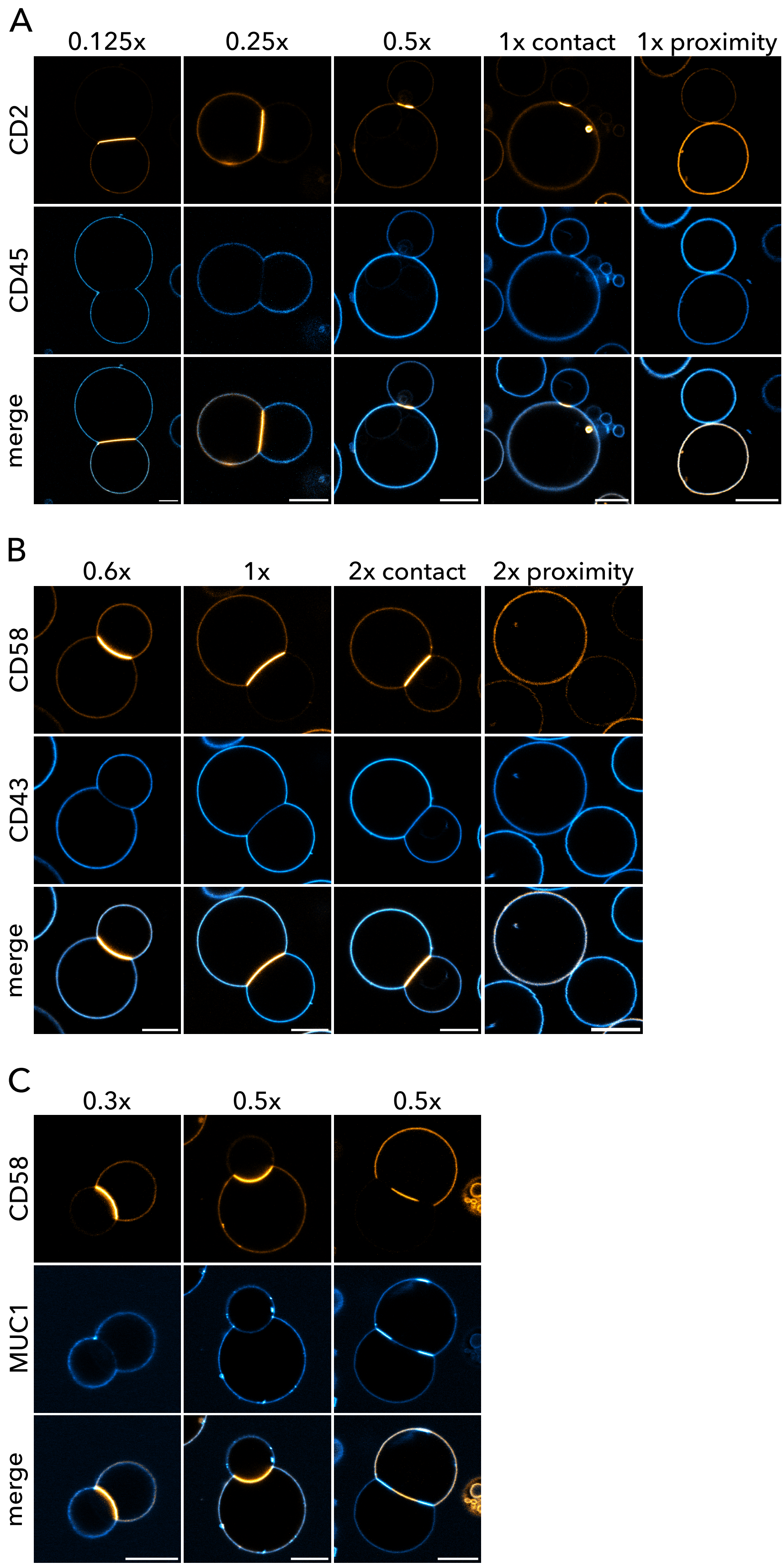

Supplement Figure 4: Exclusion of CD45, CD43, MUC1 at the CD2-CD58 contact. A| Representative confocal images of CD45-AF647 at the CD2-AF488 and CD58 (unlabelled)

contact. CD45-AF647 was added in increasing amounts (0.125x, 0.25x, 0.5x and 1x). B| Representative confocal images of CD43-AF488 at the CD58-AF647 and CD2 (unlabelled) contact. CD43-AF488 was added in increasing amounts (0.6x, 1x and 2x). C| Representative confocal images of MUC1-AF488 at the CD58-AF647 and CD2 (unlabelled) contact. MUC1-AF488 was added in increasing amounts (0.3x and 0.5x). Scale bar corresponds to 10 μm.

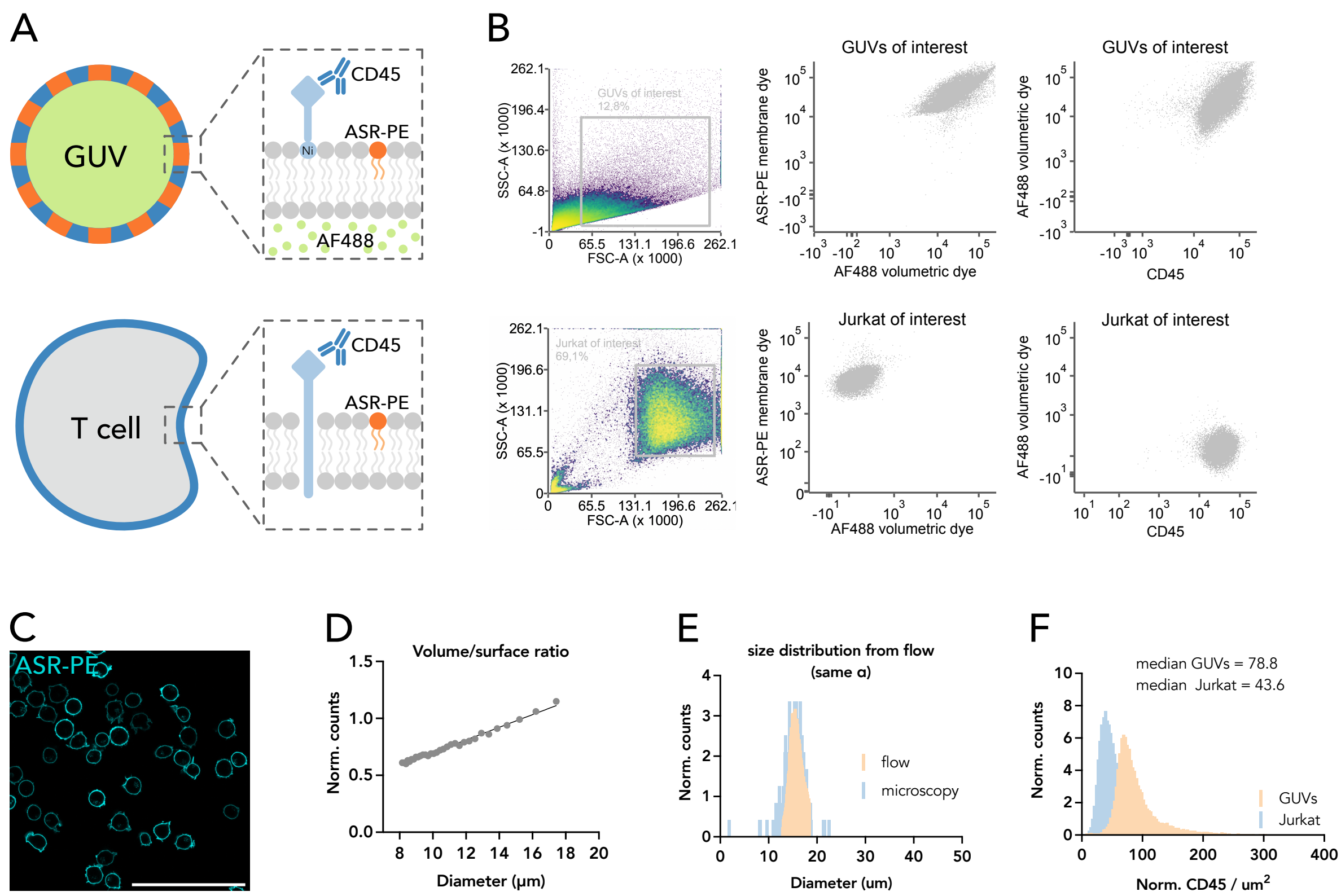

Supplement Figure 5: CD45 density estimation on GUVs and Jurkat T cells. A| Schematic overview of GUV and Jurkat T cell staining with a volumetric dye (AF488), membrane dye (ASR-PE) and anti-CD45 antibody. B| Flow cytometry plots showing the gating performed on FSC and SSC and the intensities for the respective dyes. C| Representative confocal image of Jurkat T cells used for size estimation. Scale bar = 100 μm. D| Intensity ratio of volumetric and surface dyes in relation to GUV size. E| Size distribution of Jurkat T cells estimated from microscopy and flow cytometry data. F| Distribution of normalized CD45 intensity per μm^2 for GUVs and Jurkat T cells.

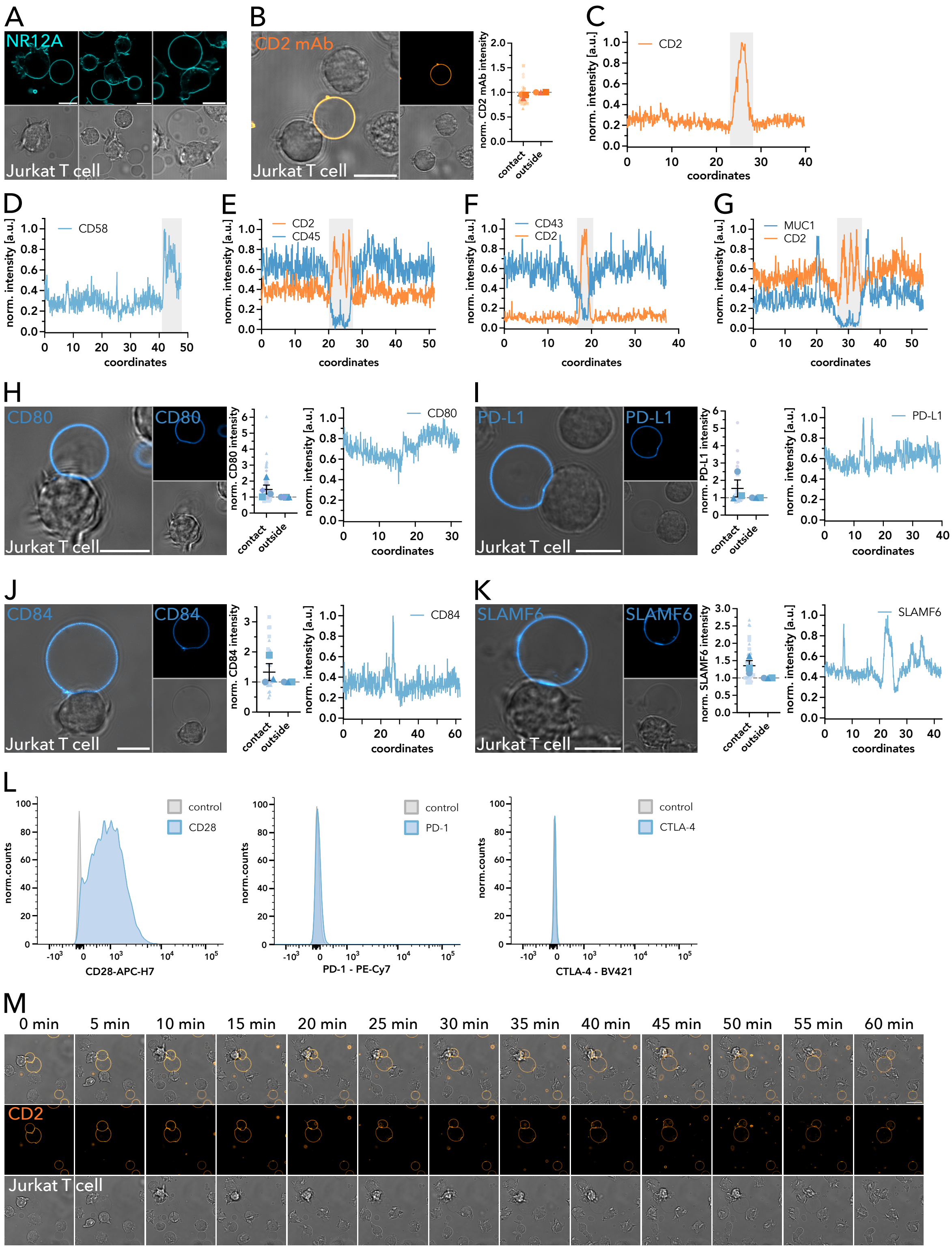

Supplement Figure 6: A| Representative confocal images of Jurkat T cells and GUVs comprising Ni-lipid stained with 100 nM NR12A. B| GUVs decorated with unlabelled CD2 and a mAb anti-CD2-AF488 was added to block CD2. Representative confocal images of these GUVs and Jurkat T cells (left). Anti-CD2-AF488 mAb intensity at the potential contact and outside was quantified and normalized (right). C-D| Linearized fluorescence intensity profiles of the GUVs decorated with either CD2-AF488 (C) or CD58-AF647 (D) with the contact

highlighted in grey in the confocal images of Figure 2 B-C. E-G| Linearized fluorescence intensity profiles of the GUVs decorated with CD2-AF488 and CD45-AF647 (E); CD2-AF647 and CD43-AF488 (F); or CD2-AF647 and MUC1-AF488 (G) with the contact highlighted in grey in the confocal images of Figure 2 F-H. H-K| Representative confocal images of contact formation between GUVs decorated with CD80 (H), PD-L1 (I), CD84 (J) or SLAMF6 (K) with Jurkat T cells (left). CD80-AF647, PD-L1-AF647, CD84-AF647 and SLAMF6-AF647 intensity at the contact and outside was quantified and normalized (middle). Linearized fluorescence intensity profiles of the GUVs in the confocal image (right). L| Flow cytometry histograms showing the expression of CD28 (left), PD-1 (middle) and CTLA-4 (right) in Jurkat T cells compared to the unstained control. M| Time-lapse of Jurkat T cells in contact with a GUV reconstituted with CD2-AF488 for up to 1 h. Superplots show individual contacts as small symbols. Large symbols depict the mean of the individual biological replicates. Symbol corresponds to the individual biological replicate ($n \geq 3$). Standard error of the mean is shown. Scale bar corresponds to 10 μm.

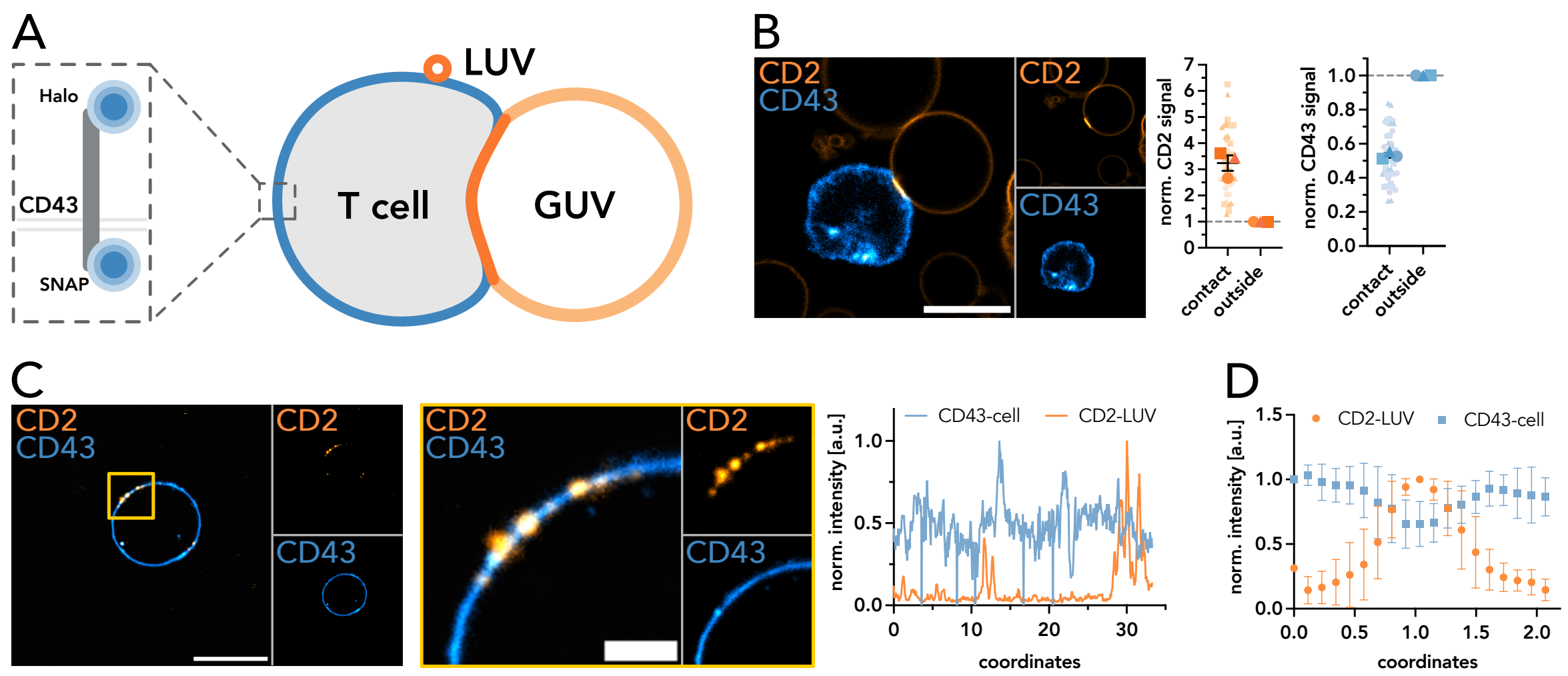

Supplement Figure 7: A| Schematic overview of CEM cells endogenously expressing CD43 with an intracellular SNAP- and extracellular Halo-tag, which interact with GUVs or LUVs reconstituted with CD2. B| Representative confocal images of contact formation between GUVs decorated with CD2 and CEM $CD43_{SNAP/Halo}$ cells stained with SiR-SNAP-substrate (left). CD2-AF488 and CD43-SiR intensity at the contact and outside was quantified and normalized (right). Superplots show individual contacts as small symbols. Large symbols depict the mean of the individual biological replicates. Symbol corresponds to the individual biological replicate (n≥3). Standard error of the mean is shown. C| Representative confocal images of contact formation between LUVs decorated with CD2 and CEM $CD43_{SNAP/Halo}$ cells stained with SiR-Halo-substrate (left). Zoomed in image on LUV-cell contact site (middle). Linearized fluorescence intensity profiles of the CEM cell with bound LUVs in the confocal image (right). n=1 D| Quantification of all LUV-CEM cell contact sites to examine CD43 distribution. Mean and standard deviation are shown. Scale bar corresponds to 10 μm and in zoom image to 2 μm.

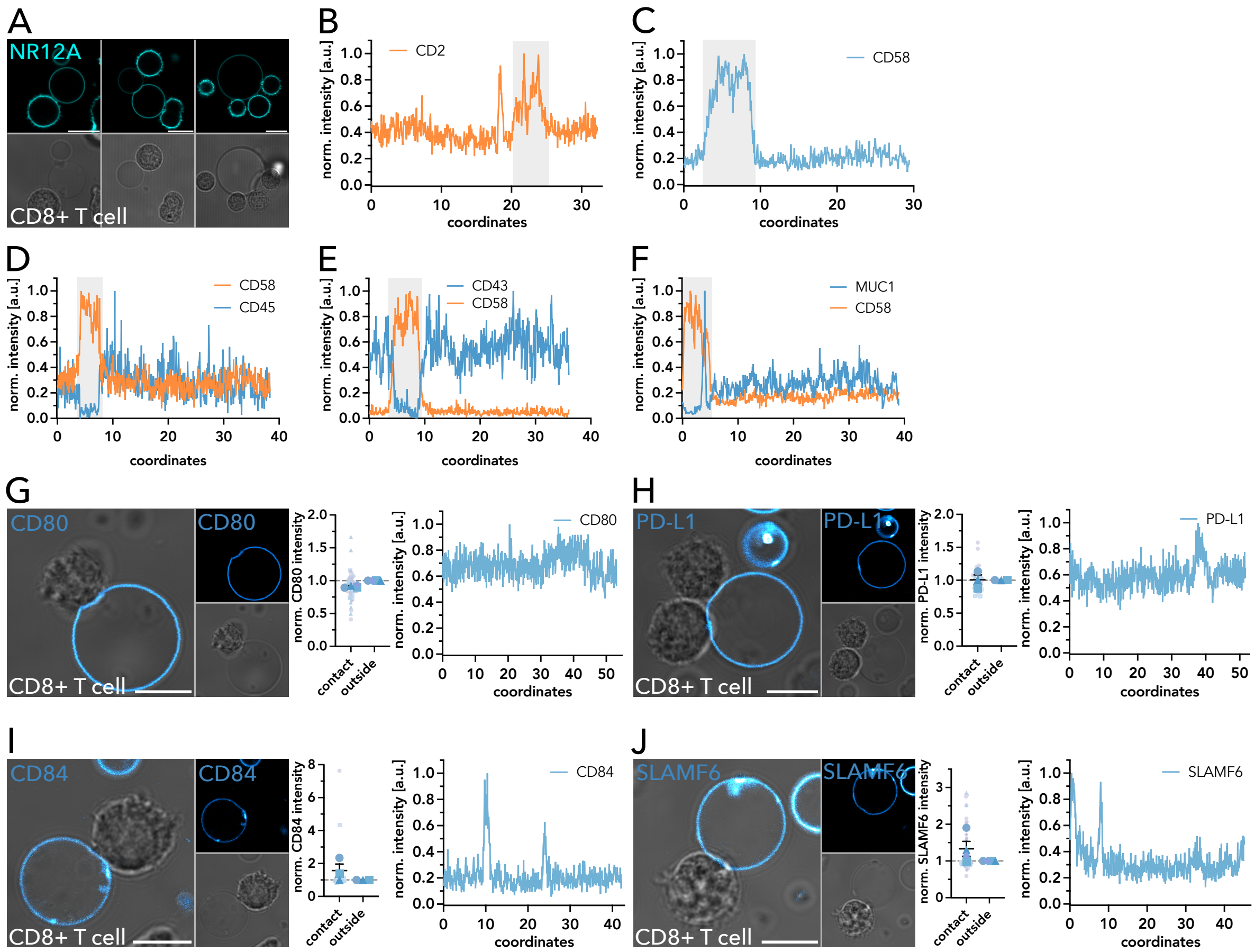

Supplement Figure 8: A| Representative confocal images of activated human primary CD8+ T cells and GUVs comprising Ni-lipid stained with 100 nM NR12A. B-C| Linearized fluorescence intensity profiles of the GUVs decorated with CD2-AF488 (B) or CD58-AF647 (C) with the contact highlighted in grey in the confocal images of Figure 2 D-E. D-F| Linearized fluorescence intensity profiles of the GUVs decorated with CD58-AF488 and CD45-AF647 (D); CD58-AF647 and CD43-AF488 (E); or CD58-AF647 and MUC1-AF488 (F) with the contact highlighted in grey in the confocal images of Figure 2 I-K. G-J| Representative confocal images of contact formation between GUVs decorated with CD80 (G), PD-L1 (H), CD84 (I) or SLAMF6 (J) with activated human primary CD8+ T cells (left). CD80-AF647, PD-L1-AF647, CD84-AF647 and SLAMF6-AF647 intensity at the contact and outside was quantified and normalized (middle). Linearized fluorescence intensity profiles of the GUVs in the confocal image (right). Superplots show individual contacts as small symbols. Large symbols depict the mean of the individual biological replicates. Symbol corresponds to the individual biological replicate (n≥3). Standard error of the mean is shown. Scale bar corresponds to 10 µm.

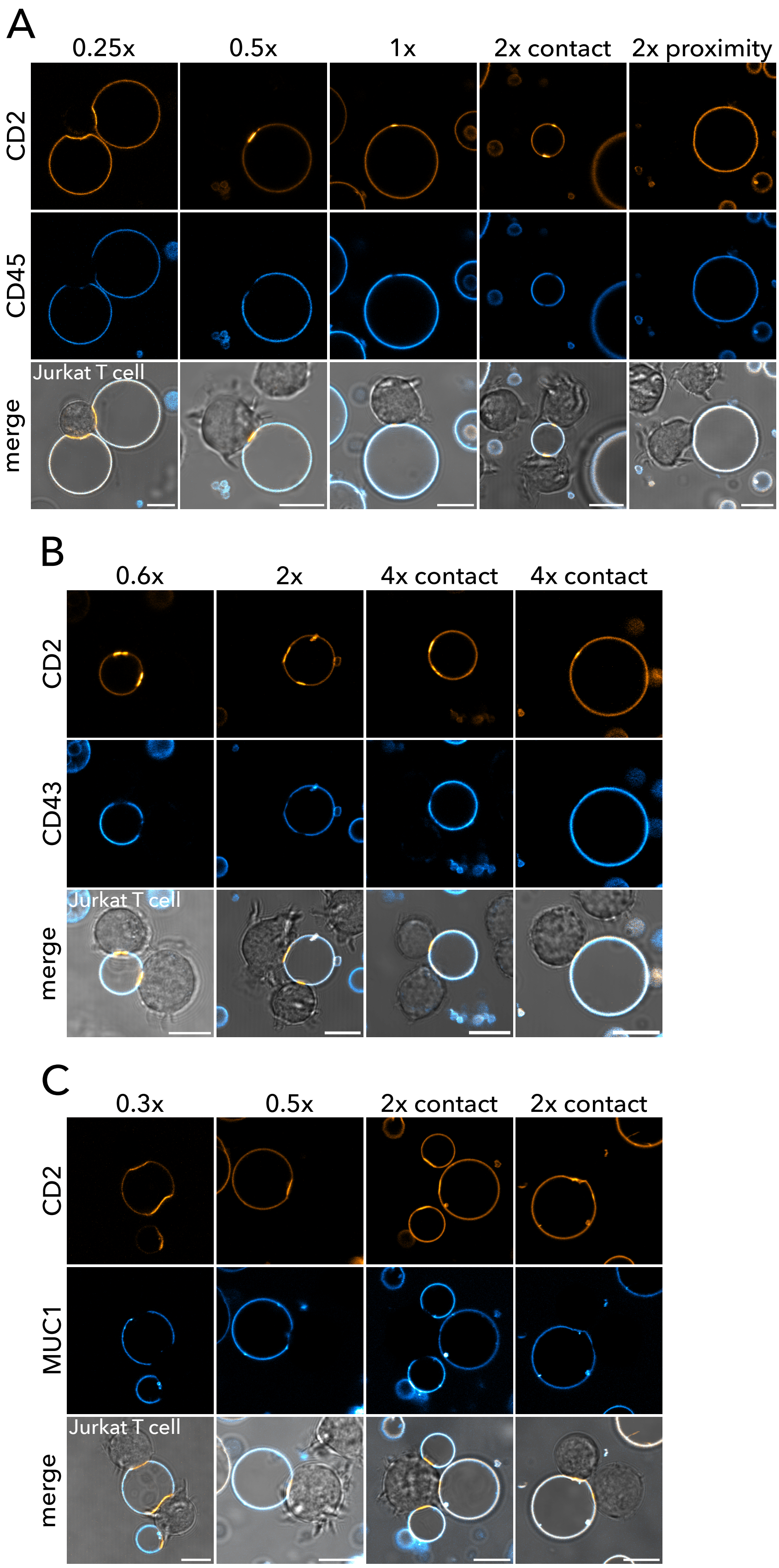

Supplement Figure 9: Exclusion of CD45, CD43, MUC1 at the CD2-decorated GUV-Jurkat T cell contact. A| Representative confocal images of contact formation between GUVs

decorated CD2-AF488 and CD45-AF647 with Jurkat T cells. CD45-AF647 was added in increasing amounts (0.25x, 0.5x, 1x and 2x). B| Representative confocal images of contact formation between GUVs decorated CD2-AF488 and CD43-AF488 with Jurkat T cells. CD43-AF488 was added in increasing amounts (0.6x, 2x and 4x). C| Representative confocal images of contact formation between GUVs decorated CD2-AF488 and MUC1-AF488 with Jurkat T cells. MUC1-AF488 was added in increasing amounts (0.3x, 0.5 and 2x). Scale bar corresponds to 10 μm.

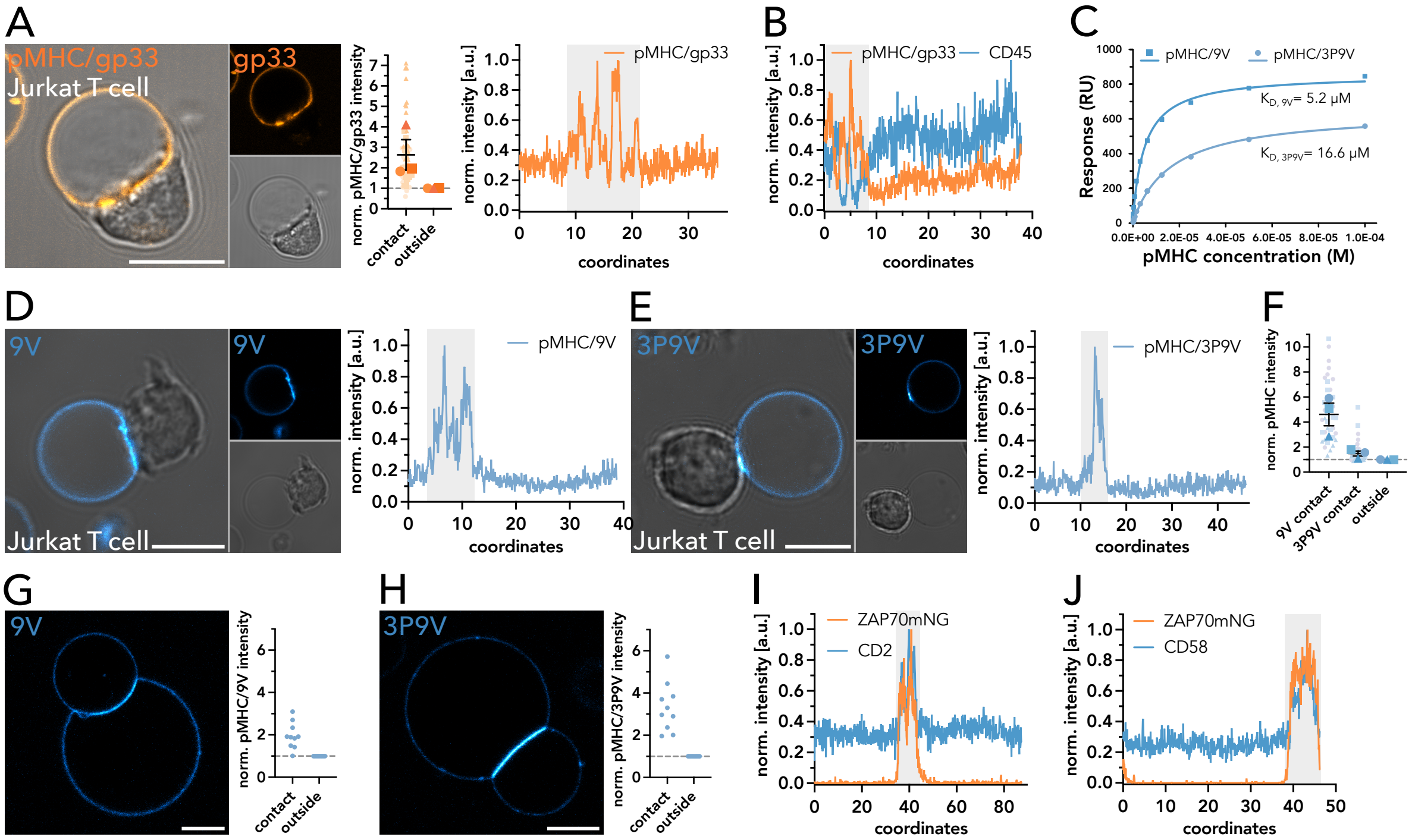

Supplement Figure 10: T cell activation.

A| Representative confocal image of contact formation between GUVs decorated with pMHC/gp33 and Jurkat P14 T cells (left). pMHC/gp33-AF488 intensity at the contact and outside was quantified and normalized (middle). Linearized fluorescence intensity profile of GUV decorated with pMHC/gp33-AF488 with the contact highlighted in grey (right). B| Linearized fluorescence intensity profile of GUV decorated with pMHC/gp33-AF488 and CD45-AF647 in the confocal images of Figure 3A. C| BIAcoreTM SPR analysis of the binding of purified 1G4 dsTCR to pMHC/9V and pMHC/3P9V. Response values were plotted against protein concentration. K_D values were obtained from steady-state fitting of equilibrium binding curves from at least ten sample injections. D-E| Representative confocal images of contact formation between GUVs decorated with pMHC/9V (D) or pMHC/3P9V (E) with Jurkat J8 T cells (left). Linearized fluorescence intensity profile of GUVs decorated with pMHC/9V-AF647 or pMHC/3P9V-AF647 with the contact highlighted in grey (right). F| Comparison of quantified and normalized pMHC/9V-AF647 and pMHC/3P9V-AF647 intensities at the contact and outside. G-H| Single protein controls. Representative confocal images of GUVs comprising Ni-lipid and with pMHC/9V (G) or pMHC3P9V-AF647 (H) (left). Fluorescent intensity at the potential contact and outside was quantified and normalized (right). I-J| Linearized fluorescence intensity profiles of the GUVs decorated with CD2-AF647 (I) or CD58-AF647 (J) in the confocal images in Figure 3D with the contact highlighted in grey. Superplots show individual contacts as small symbols. Large symbols depict the mean of the individual biological replicates. Symbol corresponds to the individual biological replicate (n=3). Standard error of the mean is shown. Scale bar corresponds to 10 µm.

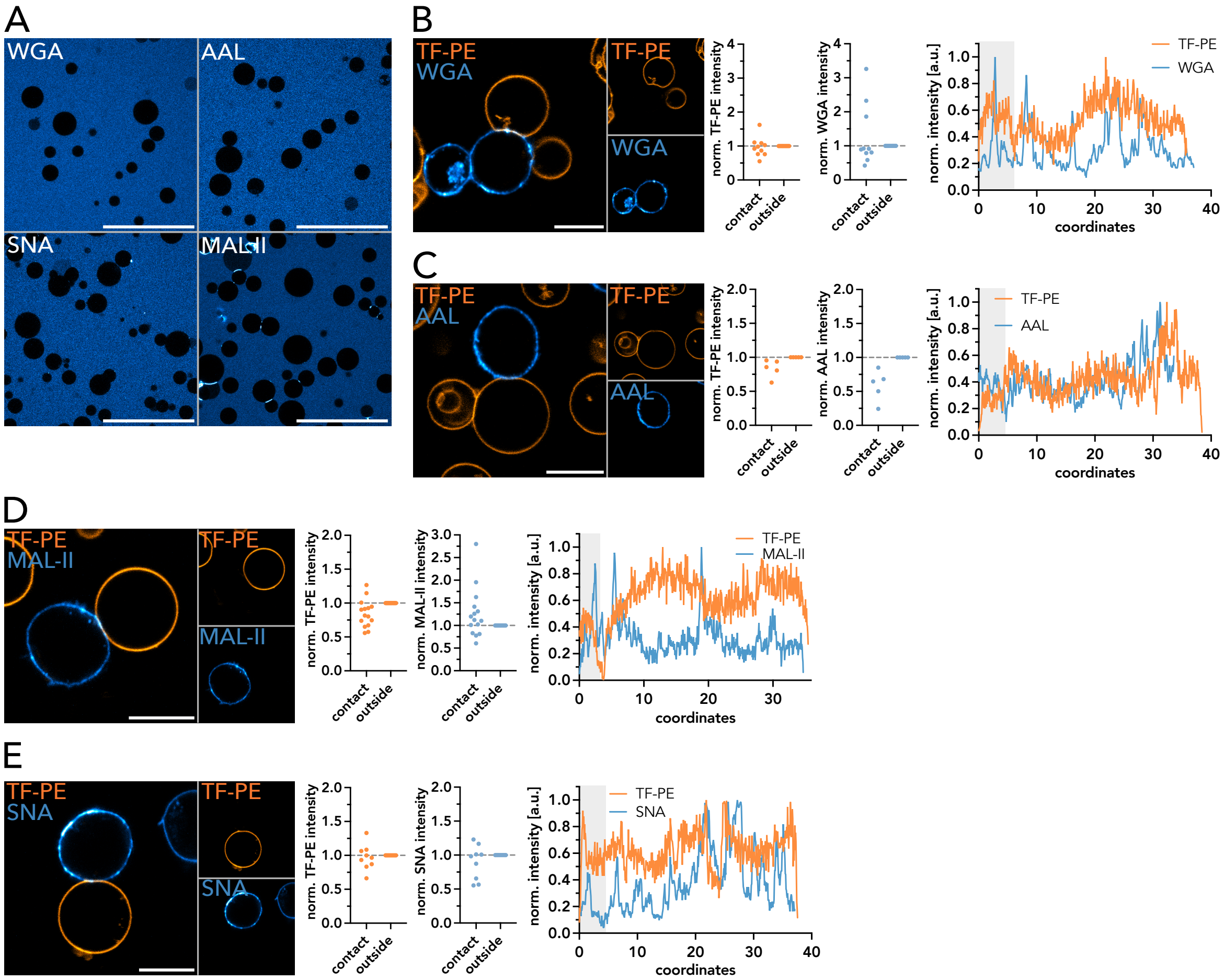

Supplement Figure 11: Glycocalyx at the T cell GUV contact. A| Lectin binding controls to no protein-GUVs containing Ni-lipid of WGA-AF647, AAL-AF647, SNA-AF647and MAL-II-AF647. B-E| Representative confocal images of contact formation between no protein-GUVs containing Ni-lipid stained with TF-PE with Jurkat T cells incubated with WGA (B), AAL (C), MAL-II (D) and SNA (E) (left). TF-PE intensity and Lectin intensity at the contact and outside were quantified and normalized (middle). Linearized fluorescence intensity profiles of the GUV and cell stained with the respective lectin in the confocal image with the contact highlighted in grey (right). Superplots show individual contacts as small symbols. Large symbols depict the mean of the individual biological replicates. Symbol corresponds to the individual biological replicate (n=3). Standard error of the mean is shown. Scale bar corresponds to 10µm.
